# Meta-analysis of sex-specific genome-wide association studies

**DOI:** 10.1002/gepi.20540

**Published:** 2010-11-18

**Authors:** Reedik Magi, Cecilia M Lindgren, Andrew P Morris

**Affiliations:** Wellcome Trust Centre for Human Genetics, University of OxfordOxford, United Kingdom

**Keywords:** genome-wide association study, meta-analysis, sex-specific effects, heterogeneity, gene-sex interaction

## Abstract

Despite the success of genome-wide association studies, much of the genetic contribution to complex human traits is still unexplained. One potential source of genetic variation that may contribute to this “missing heritability” is that which differs in magnitude and/or direction between males and females, which could result from sexual dimorphism in gene expression. Such sex-differentiated effects are common in model organisms, and are becoming increasingly evident in human complex traits through large-scale male- and female-specific meta-analyses. In this article, we review the methodology for meta-analysis of sex-specific genome-wide association studies, and propose a sex-differentiated test of association with quantitative or dichotomous traits, which allows for heterogeneity of allelic effects between males and females. We perform detailed simulations to compare the power of the proposed sex-differentiated meta-analysis with the more traditional “sex-combined” approach, which is ambivalent to gender. The results of this study highlight only a small loss in power for the sex-differentiated meta-analysis when the allelic effects of the causal variant are the same in males and females. However, over a range of models of heterogeneity in allelic effects between genders, our sex-differentiated meta-analysis strategy offers substantial gains in power, and thus has the potential to discover novel loci contributing effects to complex human traits with existing genome-wide association data. *Genet. Epidemiol*. 34:846–853, 2010. © 2010 Wiley-Liss, Inc.

## INTRODUCTION

Genome-wide association studies (GWAS) have proved to be extremely successful in mapping novel loci contributing effects to complex human traits. GWAS genotyping products are strongly biased toward common genetic variation, and are typically analyzed on a single-SNP basis. As a result, GWAS are well powered to identify common variants associated with the trait that have moderate marginal allelic effects across the population (s) under investigation. Through the efforts of large-scale international consortia, meta-analysis of GWAS from closely related populations, with effective sample sizes of tens of thousands of individuals, continue to locate additional associated common variants with ever more modest allelic effect [Barrett et al., 2009; Debette et al., 2010; Dupuis et al., 2010; Lindgren et al., 2009; Newman et al., 2010; Prokopenko et al., 2009; Stahl et al., 2010]. However, despite these successes, much of the genetic component of the variance in most complex traits remains unexplained [Manolio et al., 2009; McCarthy et al., 2008].

One potential source of genetic variation that may contribute to the “missing heritability” of complex traits is that which has sex-specific or sex-differentiated effects. In principal, sex can be thought of as a (near) perfectly measured “environmental” risk factor, which incorporates established anatomical, physiological, and behavioral differences between males and females at different stages of life [Ober et al., 2008]. As such, it is conceivable that sex could interact with causal variants, resulting in allelic effects that differ between males and females. Recent examples of confirmed sex-differentiated effects identified through human GWAS include schizophrenia with SNPs in *RELN* [Shifman et al., 2008], serum uric acid concentrations with SNPs in *SLC2A9* [Döring et al., 2008], and waist-hip ratio with SNPs in *LYPLAL1* [Lindgren et al., 2009]. Such associations could arise as a result of sexual dimorphism in gene expression, and highlight the potential for male- and female-specific GWAS to further our understanding of the etiology of complex traits.

Despite mounting evidence for sex-specific associations with complex human traits, males and females are typically analyzed together in GWAS. In these “sex-combined” analyses, allelic effects are often adjusted for gender if the distribution of the trait varies between males and females. However, researchers have been unwilling to undertake male- and female-specific analyses because of an expectation of a loss in power because of reduced sample size as a result of stratification by sex. This power loss can be partly recovered by combining the results of male- and female-specific GWAS through meta-analysis. In this article, we review the methodology for sex-specific fixed-effects meta-analysis of GWAS. Within this framework, we propose a sex-differentiated test of association, and demonstrate how we can test for heterogeneity of allelic effects between males and females. We then perform detailed simulations to evaluate the loss of power for sex-differentiated compared to sex-combined meta-analysis when allelic effects are homogenous in males and females, and the gain in power over a range of models of heterogeneity between genders.

## MODEL AND METHODS

### FIXED-EFFECTS META-ANALYSIS FRAMEWORK

Consider the results of a series of *N* sex-specific GWAS. We denote by *b*_*ij*_ and *s*_*ij*_ the allelic effect (log-odds ratio in the context of a dichotomous trait) and corresponding standard error, respectively, of the *i*th study at the *j*th SNP. We denote the sex of the *i*th GWAS by κ_*i*_, taking the value 1 if the study is male-specific and 0 otherwise.

In a fixed-effect meta-analysis framework, we obtain sex-specific allelic effect estimates, *B*_*Mj*_ and *B*_*Fj*_, in males and females, respectively, at the *j*th SNP by weighting by the inverse of the variance. Specifically,


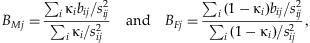


with variances given by 
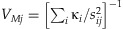
 and 
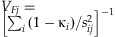
, respectively.

We can perform sex-specific tests of association across all studies at the *j*th SNP, given by 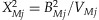 and 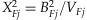, respectively, in males and females, where each of these statistics has a chi-squared distribution with one degree of freedom. We can also test for the presence of heterogeneity between studies of the same sex by means of Cochran's Q-statistic, given by


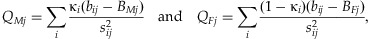


respectively, in males and females. These test statistics have chi-squared distributions with *n*_*M*_−1 and *n*_*F*_−1 degrees of freedom, respectively, where 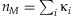 and 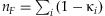.

We can perform a sex-differentiated test of association across all studies at the *j*th SNP, allowing for different allelic effects in males and females, given by 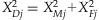, and having a chi-squared distribution with two degrees of freedom. In addition, we can test for heterogeneity between sex-specific allelic effects at the *j*th SNP by means of the test statistic 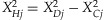, having a chi-squared distribution with one degree of freedom. In this expression, 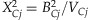 is a test of association at the *j*th SNP over all *N* sex-specific GWAS, assuming the same allelic effect in males and females. The sex-averaged allelic effect estimate over all studies is given by


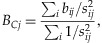


with variance 
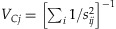
.

The methodology described above has been implemented in the GWAMA software [Magi and Morris, 2010] with use of the “–sex” option, and is freely available for download from the website http://www.well.ox.ac.uk/GWAMA. The open-source software has been designed to efficiently handle the meta-analysis of genetic association data on a genome-wide scale and incorporates a variety of error trapping facilities. The software is distributed with scripts that allow simple formatting of files containing the results of each GWAS and generate graphical summaries of genome-wide meta-analysis results.

### SIMULATION STUDY

Consider meta-analysis of 10 GWAS of a quantitative trait, each consisting of 1,000 males and 1,000 females. We perform simulations to evaluate the power of the following meta-analysis strategies:

- MALE-SPECIFIC. Analyze males only in each GWAS. Combine allelic effect estimates in a fixed-effects meta-analysis, weighted by the inverse variance, and test for association with the trait using 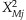.
- FEMALE-SPECIFIC. Analyze females only in each GWAS. Combine allelic effect estimates in a fixed-effects meta-analysis, weighted by the inverse variance, and test for association with the trait using 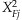.
- SEX-DIFFERENTIATED. Analyze males and females separately in each GWAS. Obtain male- and female-specific allelic effect estimates in a fixed-effects meta-analysis, and test for association with the trait, allowing for sex-differentiation using 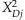.
- HETEROGENEITY. Analyze males and females separately in each GWAS. Obtain male- and female-specific allelic effect estimates in a fixed-effects meta-analysis, and test for heterogeneity between the sexes using 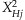.
- SEX-COMBINED. Analyze males and females combined in each GWAS of 2,000 individuals, ambivalent to sex. Combine allelic effect estimates in a fixed-effects meta-analysis, weighted by the inverse variance, and test for association with the trait.

We examine a range of models of sex-specific and sex-differentiated association with the trait, parameterized in terms of the causal allele frequency, *q*, and additive allelic effects in males and females, β_*M*_ and β_*F*_, respectively. Specifically, we consider a model of homogeneity (β_*M*_ = β_*F*_) as well as three models of heterogeneity: (i) male-specific effect (β_*F*_ = 0); (ii) same direction effects (β_*F*_ = 2β_*M*_); and (iii) opposite direction effects (β_*F*_ = −β_*M*_). Assuming equal frequencies of males and females within a population, the proportion of phenotypic variance explained by the causal variant is given by λ = *V*_*G*_/ (*V*_*G*_+*V*_*E*_), where *V*_*E*_ is the residual variance and 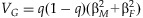. Table I summarizes the range of models considered in our simulations, together with the proportion of phenotypic variance explained by a causal variant with 50% frequency and a residual variance, *V*_*E*_ = 1, in each case. We also investigate the impact of causal allele frequency variation between the 10 GWAS, which may occur as a result of ascertainment from different populations, for example, commonly measured by means of *F*_*ST*_, denoted by *f*. In this setting, the causal allele frequency in each of the GWAS will be generated at random from a Beta (*q* (1 − *f*) /*f*, (1−*q*) (1−*f*) /*f*) distribution, according to the Balding-Nichols model [Balding and Nichols, 1995].

**Table I:** Summary of models of sex-specific and sex-differentiated allelic effects considered in the simulation study

Model	Homogeneous effects		Heterogeneous effects: male-specific		Heterogeneous effects: same direction		Heterogeneous effects: opposite directions	
				
β_*M*_	β*_F_*	λ (%)	β*_F_*	λ (%)	β*_F_*	λ (%)	β*_F_*	λ (%)
0.01	0.01	0.005	0	0.002	0.02	0.012	−0.01	0.005
0.02	0.02	0.020	0	0.010	0.04	0.050	−0.02	0.020
0.03	0.03	0.045	0	0.022	0.06	0.112	−0.03	0.045
0.04	0.04	0.080	0	0.040	0.08	0.200	−0.04	0.080
0.05	0.05	0.125	0	0.062	0.10	0.312	−0.05	0.125
0.06	0.06	0.180	0	0.090	0.12	0.448	−0.06	0.180
0.07	0.07	0.244	0	0.122	0.14	0.609	−0.07	0.244
0.08	0.08	0.319	0	0.160	0.16	0.794	−0.08	0.319
0.09	0.09	0.403	0	0.202	0.18	1.002	−0.09	0.403
0.10	0.10	0.498	0	0.249	0.20	1.235	−0.10	0.498

The parameters β*_M_* and β*_F_* denote the male- and female-specific allelic effects, respectively. For each model, the contribution of a causal variant of 50% frequency to the overall phenotypic variance, denoted λ, is also presented, assuming equal frequencies of males and females in each population and a residual variance of 1.

For each model, we simulate 10,000 replicates of genotype and phenotype data for the 1,000 males and 1,000 females in each of the 10 GWAS. For each individual, the genotype is simulated on the basis of the causal allele frequency, *q*, under an assumption of Hardy-Weinberg equilibrium. Conditional on this genotype, the phenotype is then generated from a Gaussian distribution with unit variance, and mean given by the appropriate sex-specific effect, β_*M*_ or β_*F*_, according to an additive model. Sex-specific and sex-combined analyses are performed within each cohort in a linear regression modeling framework, assuming additive allelic effects. Maximum-likelihood estimates of the male- and female-specific, and sex-combined allelic effects, and their corresponding standard errors, are obtained, and combined across studies using the five fixed-effects meta-analysis strategies described above. Over all replicates, false-positive error rates are estimated at a nominal *P* = 5 × 10^−2^ significance threshold (for models in which there is no effect in either sex, β_*M*_ = β_*F*_ = 0), while power is estimated at a stringent genome-wide significance threshold of *P* = 10^−8^. For each model, we also obtain the mean square error of the male- and female-specific allelic effect estimates, *B*_*M*_ and *B*_*F*_, defined above, together with that for the sex-combined analysis, by comparison to the true effect sizes, β_*M*_ and β_*F*_.

## RESULTS

Table II presents the false-positive error rate, at a *P* = 5 × 10^−2^ significance threshold, for each meta-analysis strategy, as a function of overall allele frequency and *F*_*ST*_. The range of *F*_*ST*_ considered here encompass no differences in allele frequencies between GWAS (*F*_*ST*_ = 0), to the extent of differences expected between GWAS in populations from different ethnic groups (*F*_*ST*_ = 0.1). Irrespective of overall allele frequency and *F*_*ST*_, the false-positive error rate of each meta-analysis strategy is entirely consistent with a *P* = 5 × 10^−2^ significance threshold, suggesting population differences between GWAS will not result in anti-conservative tests of association.

**II:** False-positive error rates (%), at a *P* = 5 × 10^−2^ significance threshold, for five meta-analyses strategies as a function of allele frequency and *F_ST_*

		Meta-analysis strategy				
		
Allele frequency	*F_ST_*	Sex-combined	Male-specific	Female-specific	Sex-differentiated	Heterogeneity
0.1	0	4.91	5.19	5.21	5.25	5.24
	10^−4^	5.17	4.80	5.24	4.82	4.62
	10^−3^	4.94	5.13	5.00	5.00	4.66
	10^−2^	4.70	4.62	4.96	4.84	4.63
	10^−1^	5.07	5.22	5.09	5.05	5.06
0.2	0	4.83	5.09	4.50	4.81	4.86
	10^−4^	4.97	4.92	4.94	4.82	4.68
	10^−3^	4.93	5.38	4.73	4.91	5.02
	10^−2^	5.27	5.33	5.22	5.30	5.26
	10^−1^	5.00	4.94	5.05	5.03	4.90
0.5	0	5.19	5.30	5.02	5.27	4.87
	10^−4^	5.23	5.04	5.40	5.22	5.36
	10^−3^	5.43	5.47	5.05	5.43	5.18
	10^−2^	4.97	5.06	5.10	5.27	4.94
	10^−1^	5.06	5.35	5.18	5.38	5.54

Rates are estimated over 10,000 replicates of meta-analysis of 10 GWAS of 1,000 males and 1,000 females.

**Fig. 1:**
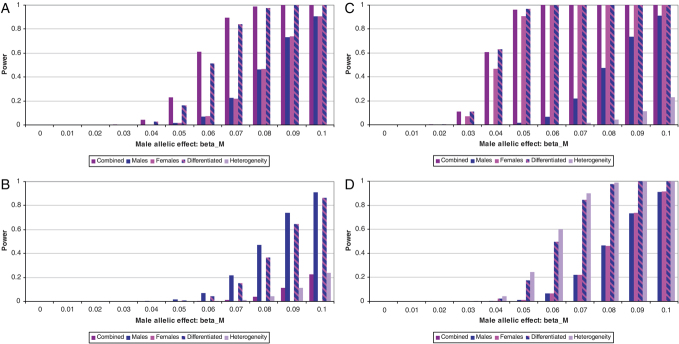
Power of five meta-analysis strategies (genome-wide significance threshold of *P* = 10^−8^) for the detection of association with a causal variant (50% allele frequency) as a function of the allelic effect, β_*M*_, in males. The four panels correspond to different models of female-specific allelic effects, summarized in Table I: (A) homogeneity across males and females (β_*F*_ = β_*M*_); (B) no effect in females (β_*F*_ = 0); (C) heterogeneity between males and females in the same direction (β_*F*_ = 2β_*M*_); and (D) heterogeneity between males and females in the opposite direction (β_*F*_ = −β_*M*_). Power is estimated over 10,000 replicates of meta-analysis of 10 GWAS of 1,000 males and 1,000 females. GWAS, genome-wide association studies.

**Fig. 2:**
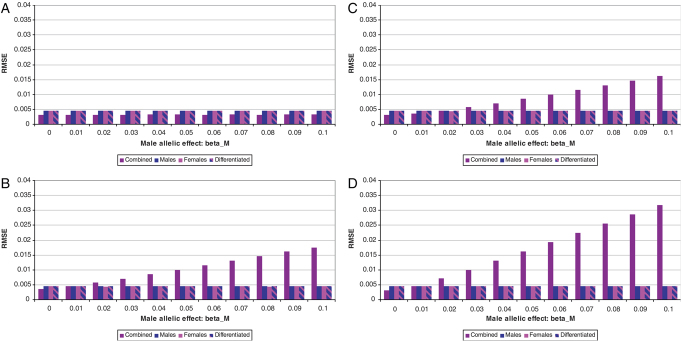
RMSE of male- and female-specific allelic effect estimates at a causal variant with (50% allele frequency) for four meta-analysis strategies as a function of the allelic effect, β_*M*_, in males. The four panels correspond to different models of female-specific allelic effects, summarized in Table I: (A) homogeneity across males and females (β_*F*_ = β_*M*_); (B) no effect in females (β_*F*_ = 0); (C) heterogeneity between males and females in the same direction (β_*F*_ = 2β_*M*_); and (D) heterogeneity between males and females in the opposite direction (β_*F*_ = −β_*M*_). RMSE is estimated over 10,000 replicates of meta-analysis of 10 GWAS of 1,000 males and 1,000 females. RMSE, root mean square error; GWAS, genome-wide association studies.

Figure 1 presents the power to detect association, at a stringent genome-wide significance threshold of *P* = 10^−8^, for each meta-analysis strategy, for a causal variant with 50% frequency, with all GWAS ascertained from the same population (*F*_*ST*_ = 0). Results are presented as a function of the male-specific allelic effect. Figure 2 presents the root mean square error (RMSE) of male- and female-specific allelic effect estimates at the causal variant for the same range of models. As described above, the four panels in each figure correspond to different models of female-specific allelic effects, summarized in Table I: (a) homogeneity across males and females (β_*F*_ = β_*M*_); (b) no effect in females (β_*F*_ = 0); (c) heterogeneity between males and females in the same direction (β_*F*_ = 2β_*M*_); and (d) heterogeneity between males and females in the opposite direction (β_*F*_ = −β_*M*_).

In the absence of heterogeneity of allelic effects between males and females, meta-analysis of sex-combined GWAS provides the greatest power to detect association with the causal variant. The loss in power of the sex-differentiated analysis is not overwhelming, and occurs as a result of the additional degree of freedom required to allow for heterogeneity between male- and female-specific allelic effects. The male- and female-specific meta-analyses lack power compared with these two strategies because they are each based on just half the sample size of the sex-combined and sex-differentiated meta-analyses. As expected, the heterogeneity test has no power to detect differences in allelic effects between males and females. There is little difference in RMSE between the meta-analysis strategies, with all providing equally precise estimates of male- and female-specific effects.

For a model of male-specific association, there is no power to detect association with the causal variant in females, as expected. The most powerful approach under this model is meta-analysis of male-specific GWAS. However, the loss in power of the sex-differentiated analysis is, again, not overwhelming, and here represents inclusion of females that provide no additional information about association, and the additional degree of freedom to allow for heterogeneity in allelic effects between genders. The meta-analysis of sex-combined GWAS is noticeably less powerful than these two strategies, despite the increase in sample size compared with male-specific GWAS, because of a weakening of the overall allelic effect by the inclusion of females. As before, there is little difference in RMSE between the sex-specific and sex-differentiated meta-analyses. However, the allelic estimates from meta-analysis of sex-combined GWAS are noticeably less precise: male effects are under-estimated, whilst those in females are over-estimated.

In the presence of heterogeneity in allelic effects between the sexes, with the direction of effect in males and females being the same, sex-differentiated meta-analysis offers greatest power to detect association with a causal variant. There is a negligible loss of power for meta-analysis of sex-combined GWAS, representing a trade-off between the extent of heterogeneity in allelic effects between genders, and the additional degree of freedom required to allow for this difference. The sex-specific meta-analyses are noticeably less powerful than these two strategies because they are based on smaller sample sizes. Of these, the power of the male-specific meta-analysis is lowest because the allelic effect is greater in females. As before, allelic estimates from meta-analysis of sex-combined GWAS are noticeably less precise than from any of the other strategies, this time because female effects are under-estimated, while those in males are over-estimated.

In the presence of heterogeneity in allelic effects between the sexes, with the opposite direction of effect in males and females, there is no power to detect association with the causal variant through meta-analysis of sex-combined GWAS. With this strategy, male and female allelic effects are effectively canceled out within each study by analyzing both sexes together. Sex-differentiated meta-analysis offers substantially greater power than either of the sex-specific meta-analyses, representing a trade-off in sample size against the additional degree of freedom to model heterogeneity between males and females. Given the extreme nature of differences in male and female effects under this model, there is greater power to detect heterogeneity here than when the direction of effect is the same in the two genders, despite the lower overall contribution of the causal variant to the phenotypic variance (Table I). As before, allelic estimates from meta-analysis of sex-combined GWAS are noticeably less precise than from any of the other strategies. Under this model, the sex-combined analysis will estimate the allelic effect estimate at approximately zero, with the result that female effects are over-estimated, while those in males are under-estimated.

We have also investigated the impact of *F*_*ST*_ between GWAS on the power of each of the five meta-analysis strategies. Our results (not presented) demonstrate that *F*_*ST*_ has no impact on the *relative* performance of the five strategies in terms of power and RMSE for the four models of homogeneity and heterogeneity of male- and female-specific allelic effects that we have investigated here. For the most extreme population differences considered here, *F*_*ST*_ = 0.1, there was a small reduction in power for all strategies, but no change in RMSE.

## DISCUSSION

In this article, we have proposed a framework for meta-analysis of sex-specific GWAS to test for: (i) sex-differentiated association of SNPs with quantitative or dichotomous traits and (ii) heterogeneity of allelic effects between males and females. The results of our simulation study highlight only a small loss in power of our sex-differentiated approach as compared to meta-analysis of sex-combined GWAS when the allelic effects of the causal variant are the same in males and females. However, over a range of models of heterogeneity in allelic effects between genders, our sex-differentiated meta-analysis of sex-specific GWAS offers substantial gains in power. An alternative approach might simply focus on the presentation of results of male-specific, female-specific and sex-combined analyses, without formal testing of sex-differentiated effects. However, in principal, such a strategy should be penalized for multiple testing, since each SNP is analyzed three times. Furthermore, our simulations suggest that, even without correction for multiple testing, this approach will lack power compared to sex-differentiated tests of association when causal variants have allelic effects in *both* males and females, but which differ in direction and/or magnitude between them.

The sex-differentiated meta-analysis is equivalent to testing for phenotype association with SNPs allowing for interaction between genotypes and gender under an additive model. Furthermore, our approach to evaluate the evidence for heterogeneity of allelic effects between males and females is equivalent to a formal test of interaction with sex. The key advantage of our framework is the basis on meta-analysis of the results of sex-specific GWAS, implemented in the GWAMA software [Magi and Morris, 2010]. Such results are straightforward to obtain using GWAS analysis software, such as PLINK [Purcell et al., 2007] and SNPTEST [Marchini et al., 2007], and do not rely on fitting more complex interaction models within each study which may, themselves, be complicated to combine via meta-analysis.

There is mounting evidence of an important role for sex-differentiated effects in the architecture of complex traits, the most compelling of which has been observed in model organisms. For example, fear conditioning, blood pressure, and renal phenotypes all demonstrate sex-specific effects in mice [Athirakul et al., 2008; Ponder et al., 2007]. Studies in a rat model suggested evidence for sex-differentiated effects of an insertion-deletion in the *ACE* gene with hypertension, a result which has now been replicated in humans [Higaki et al., 2000; O'Donnell et al., 1998; Stankovic et al., 2002]. Sex-differentiated analyses of complex human traits in well-powered GWAS have not been so widely reported to date. In *RELN*, the strongest signal of association in a GWAS of schizophrenia in Ashkenazi Jews occurred at rs7341475, demonstrating allele frequency differences in female cases and controls (*P* = 9.8 × 10^−5^), but not in males (*P* = 4.7 × 10^−1^). The female-specific association was followed up in additional samples from four populations and, although not reaching genome-wide significance (*P*<5 × 10^−8^), demonstrated consistency of allelic effects across all studies [Shifman et al., 2008], and is a strong biological candidate for brain abnormalities [Hong et al., 2000]. In the region flanking *LYPLAL1*, SNPs including rs2605100 demonstrated moderate evidence for association with waist-hip ratio in sex-combined meta-analysis of GWAS from European ancestry populations (*P* = 3.6 × 10^−6^). However, sex-specific analyses revealed the association to be genome-wide significant in females (*P* = 1.3 × 10^−8^), but entirely absent in males (*P* = 5.0 × 10^−1^) [Lindgren et al., 2009]. *LYPLAL1* has been reported to be up-regulated in subcutaneous adipose tissue from obese subjects, and thus is a strong biological candidate for central obesity [Steinberg et al., 2007]. In a GWAS of serum uric acid concentrations, the strongest signal of association occurred at rs7442295 in *SLC2A9* and was replicated in three further independent cohorts (combined *P* = 3.0 × 10^−70^) [Döring et al., 2008]. On further inspection of sex-specific results, the association was observed to be substantially stronger in females (*P* = 2.6 × 10^−74^, explaining 5.8% of the phenotypic variance) than males (*P* = 7.0 × 10^−17^, explaining 1.2% of the phenotypic variance).

The identification of sex-differentiated associations is clearly more challenging than allelic effects that are homogeneous in males and females. However, meta-analysis of GWAS through the efforts of large-scale international consortia means that, for many traits, we are now in a position to adequately address these challenges. Sex interaction effects are common in model organisms for a wide range of traits, and often account for a substantial proportion of the genetic component of phenotypic variation [Ober et al., 2008]. Sex-differentiated analyses have not traditionally performed in GWAS, so it is difficult to assess the likely impact of sex-specific effects on complex human traits on the basis of data. However, given the extent of sex-differentiated genetic architecture in model organisms, there is no reason to believe that heterogeneity in allelic effects between males and females will not also exist in humans. In this article, we have taken the first steps in developing a unified framework to allow for meta-analysis of sex-specific GWAS, implemented in the GWAMA software. The coming months promise an exciting period of application of sex-differentiated meta-analysis to existing and future GWAS, with the potential to uncover novel loci contributing effects to complex traits, furthering our understanding of the genetic architecture underpinning these phenotypes.
